# Nonmuscle Myosin II helps regulate synaptic vesicle mobility at the *Drosophila *neuromuscular junction

**DOI:** 10.1186/1471-2202-11-37

**Published:** 2010-03-16

**Authors:** Sara Seabrooke, Xinping Qiu, Bryan A Stewart

**Affiliations:** 1 Department of Biology, University of Toronto, Mississauga, ON L5L 1C6, Canada

## Abstract

**Background:**

Although the mechanistic details of the vesicle transport process from the cell body to the nerve terminal are well described, the mechanisms underlying vesicle traffic within nerve terminal boutons is relatively unknown. The actin cytoskeleton has been implicated but exactly how actin or actin-binding proteins participate in vesicle movement is not clear.

**Results:**

In the present study we have identified Nonmuscle Myosin II as a candidate molecule important for synaptic vesicle traffic within *Drosophila *larval neuromuscular boutons. Nonmuscle Myosin II was found to be localized at the *Drosophila *larval neuromuscular junction; genetics and pharmacology combined with the time-lapse imaging technique FRAP were used to reveal a contribution of Nonmuscle Myosin II to synaptic vesicle movement. FRAP analysis showed that vesicle dynamics were highly dependent on the expression level of Nonmuscle Myosin II.

**Conclusion:**

Our results provide evidence that Nonmuscle Myosin II is present presynaptically, is important for synaptic vesicle mobility and suggests a role for Nonmuscle Myosin II in shuttling vesicles at the *Drosophila *neuromuscular junction. This work begins to reveal the process by which synaptic vesicles traverse within the bouton.

## Background

Transport and assembly of synaptic vesicles has been the subject of several studies. Vesicles and their components are transported along axon microtubules to the nerve terminal, (for review see [1,2]) where they participate in synaptic physiology, undergoing a cycle of exo- and endocytosis. However, vesicle traffic within terminal boutons is not well understood although recent advances in this area have been made [3,4].

Classically, vesicles were believed to be relatively stationary until released [5-7]. However, more recent studies provided evidence for a mobile vesicle pool [8,9] best described by a caged-diffusion model [10] and differential vesicle mobility in the reserve and recycling pool has been suggested within the frog motor nerve terminals [11]. Additionally, Nunes et al. [12] observed dynamic vesicles in the *Drosophila melanogaster *bouton, and dynamic vesicles have been reported at ribbon synapses in lizards [13].

Vesicle movement may result from diffusion or directed transport. Actin polymerization in the nerve terminal may promote vesicle movement through a *Listeria *comet mechanism [14] or it may act as a substrate for myosin motors to shuttle vesicles. In light of a previous screen from our lab identifying Nonmuscle Myosin II (NMMII) as a candidate molecule important in neuromuscular junction (NMJ) development [15,16], we have focused on determining a neuromuscular function for this actin-based myosin motor. NMMII is present in the nervous system of *Xenopus*, mouse, rat and chicken [17-19], and in the CNS of *Drosophila *[20]. NMMII can both crosslink F-actin and has been shown to transport vesicles on F-actin [21-23].

This study was undertaken to determine whether NMMII contributes to synaptic vesicle mobility. We used the genetic model system *Drosophila melanogaster *to manipulate NMMII expression, pharmacology to inhibit NMMII activity, and the optically accessible neuromuscular synapse of third instar larvae to investigate vesicle dynamics. We show that NMMII is concentrated pre- and postsynaptically and importantly, we report that unstimulated synaptic vesicle mobility exhibited a dependence on NMMII expression. These results report the first evidence for NMMII having a function in synaptic vesicle dynamics at the *Drosophila *NMJ.

## Results

### Nonmuscle Myosin II is localized pre- and postsynaptically at the NMJ

**Figure 1:**
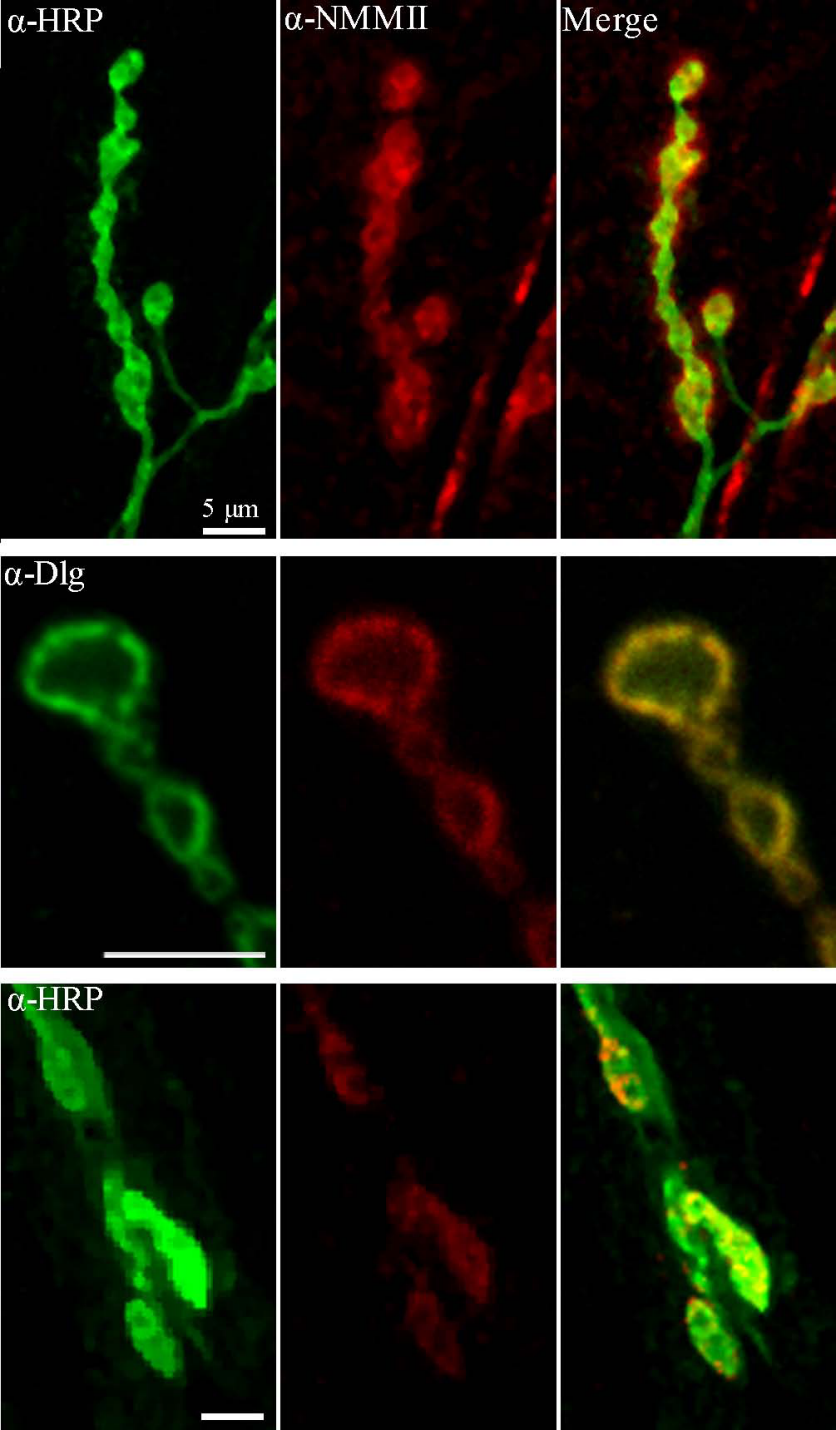
**NMMII is localized pre- and postsynaptically at the NMJ**. α-NMMII (red) was found to colocalize with the neural marker α-HRP (green). α-NMMII staining is observed which did not colocalize with α-HRP (merge) suggesting NMMII is also present postsynaptically (top row). The postsynaptic marker α-Dlg (green) is observed to colocalize (merge) with α-NMMII (red) suggesting NMMII is present postsynaptically (second row). To confirm presynaptic expression of NMMII, *UASzipRNAi *was expressed postsynaptically using *24BGal4 *(bottom row). This eliminated postsynaptic expression of NMMII. Immunocytochemistry revealed that while postsynaptic staining of NMMII had been eliminated, NMMII was still present presynaptically as seen with the colocalization with α-HRP (merge). No postsynaptic NMMII is visible in the merged image. The top and bottom rows are shown as confocal stacks, while the middle two rows are shown as single confocal slices.

Although NMMII has been previously found in the *Drosophila *CNS [20], it has not been previously localized to the NMJ. Therefore we used immunocytochemistry to investigate the localization of NMMII at the NMJ (Figure. 1). First, we demonstrated the presence of NMMII by staining the NMJ with anti-NMMII and found a robust signal labelling the nerve terminal, which co-localized to a high degree with anti-HRP, a general marker of insect neural membrane. We did notice however that some of the NMMII staining was found outside the boutons defined by the anti-HRP signal suggesting that some of the NMMII signal has a postsynaptic origin. To further investigate this possibility we double labelled the NMJ with the post-synaptic marker, Discs large and anti-NMMII. We found colocalization between Discs large and NMMII, indicating NMMII is found post-synaptically. To determine whether NMMII is also found presynaptically, we expressed *UASzipRNAi *in the muscle using the muscle specific driver *24BGal4*. This dramatically reduces NMMII in the muscle, revealing presynaptic NMMII, which colocalizes with the specific neural marker anti-HRP. Together these results demonstrate the presence of NMMII at the *Drosophila *NMJ.

### Expression level of Nonmuscle Myosin II in zipper alleles

**Figure 2:**
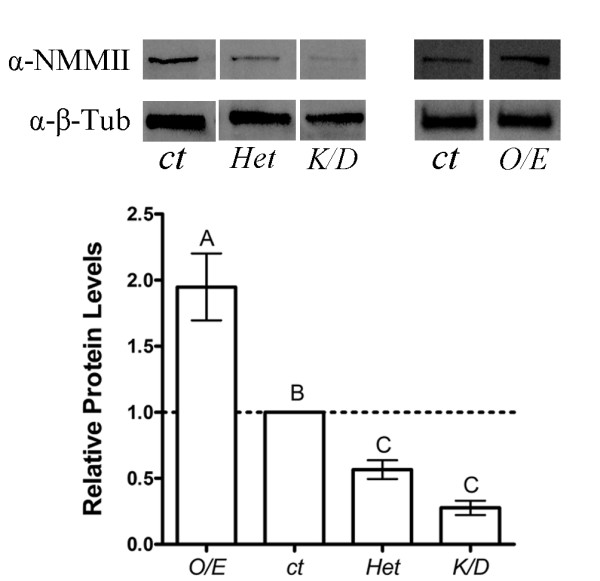
**Quantification of NMMII alleles**. Western blot of NMMII alleles *a*, Knockdown (*K/D*) of NMMII in the nervous system reduced NMMII expression to 28% (n = 5, P < 0.05) whereas the heterozygous loss-of-function *zip*^*1*^*/CyO *(*Het*) reduced NMMII expression to 57% (n = 4, P < 0.05) compared to the *elav*^*3A*^*Gal4 *control (*ct*). Overexpression of *zip*^*GS50077 *^(*O/E*) in the nervous system increased NMMII expression by 95% (n = 4, P < 0.05) compared to the *elav*^*3A*^*Gal4 *control (*ct*). α-β-Tubulin was used as the loading control. Relative NMMII proteins levels were quantified from the western blot analysis. Each band was first normalized to the level of β-tubulin (used as a loading control, not shown) and then the mutant genotypes were normalized to *elav*^*3A*^*Gal4 *protein levels, which were set to 1. The bars indicate the mean level obtained from 4-5 samples, bars are SEM. Overexpression (O/E), knockdown (K/D), heterozygous loss-of-function (Het).

In order to study NMMII we manipulated its expression level using genetic tools. In *Drosophila*, NMMII is encoded by the *zipper *(*zip*) gene. We used the heterozygous loss-of-function *zip*^*1 *^allele (*Het*), overexpressed *zip *with the *zip *transgene, *zip*^*GS50077 *^(*O/E*), and expressed a *zip *knockdown construct, *zipRNAi *(*K/D*). Before proceeding with using these reagents in experiments we confirmed the different *zip *tools were functioning as expected by western blot analysis of protein prepared from larval brain extracts (Figure. 2). NMMII expression in *elav*^*3A*^*Gal4 *(ct) larvae were used as a control and normalized to 1.00. RNAi Knockdown of NMMII significantly reduced the NMMII level to 28% (*K/D*, 0.28 ± 0.05, n = 5) of the control. The heterozygous loss-of-function allele *zip*^*1*^/*CyO *reduced NMMII expression to 57% (*Het*, 0.57 ± 0.07, n = 4) of the control while overexpression of NMMII increased protein levels by 95% compared (*O/E*, 1.95 ± 0.25, n = 4) to *elav*^*3A*^*Gal4 *(ct) (Figure 2). Statistical analysis revealed significant variation among the groups (ANOVA, p < 0.0001), while post hoc Newman-Keuls comparison between groups showed that each group was different from the other (at least p < 0.05) with the exception that *zip*^*1*^*/CyO *was not found to be statistically different from the knockdown sample despite the 50% difference in average protein levels.

### Inhibition of Myosin with ML-9 results in a dose-dependent decrease in vesicle mobility

**Figure 3:**
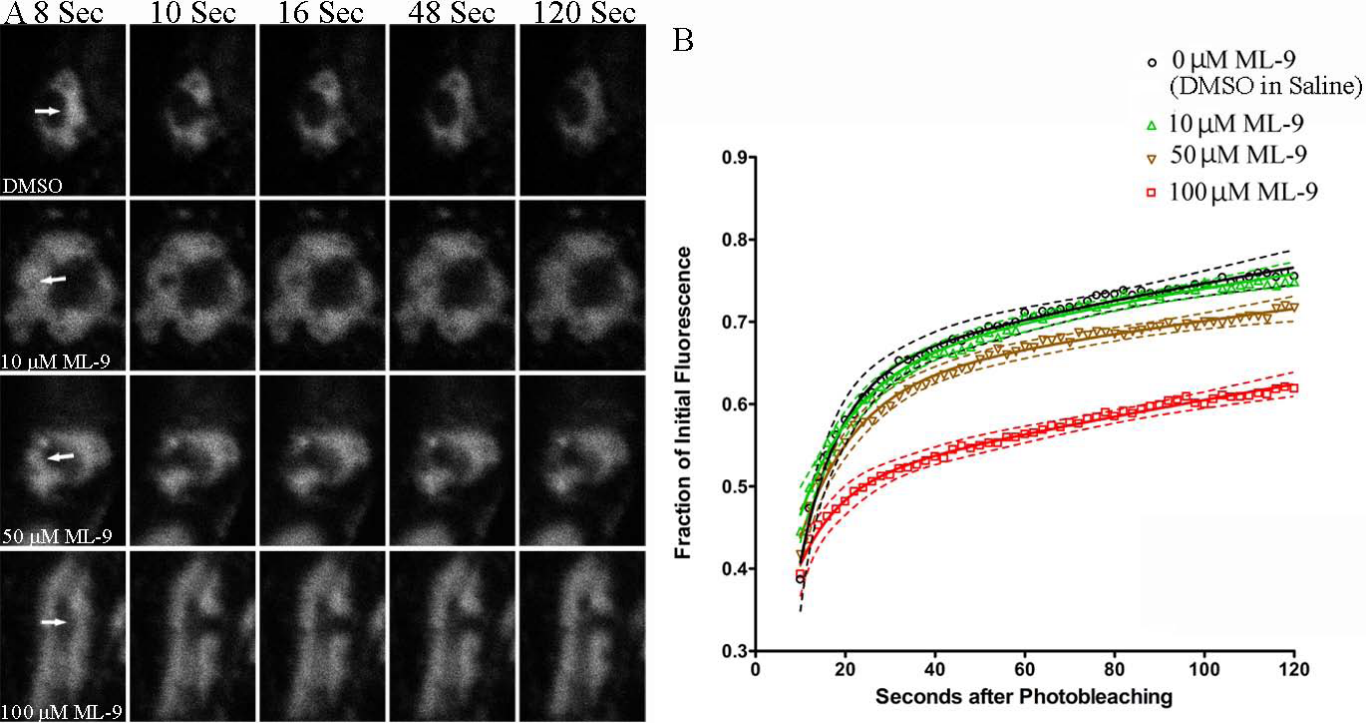
**FRAP indicating the mobility of vesicles in response to application of the Myosin inhibitor, ML-9**. *a*, Acquired images for the recovery of fluorescently labelled vesicles using *elav*^*C155*^*Gal4;UAS-sytGFP/Y *after application of ML-9. Images are shown immediately before bleaching (8 sec), immediately after photobleaching (10 sec) and at 16, 48 and 120 sec. Bleached areas are indicated by white arrows. A reduction in recovery is observed with increasing doses of ML-9. *b*, A significant dose-dependent response was observed for inhibition with ML-9 between the doses of 100 μM ML-9 and 10 μM ML-9 as compared to 0 μM ML-9. 10 μM ML-9 did not significantly reduced vesicle mobility. Error is represented as the 95% confident interval of the curve. FRAP recoveries were fit with double exponential curves and nonlinear regression was used to test for statistical differences. Sample sizes and significance were as follows: 0 μM ML-9 (n = 20), 10 uM ML-9 (n = 22, p > 0.05), 50 μM ML-9 (n = 20, p < 0.05), 100 μM ML-9 (n = 20, p < 0.05).

NMMII has been previously shown to function in NMJ development [15,16]. For this reason, we investigated whether NMMII affected vesicle dynamics at the *Drosophila *NMJ. To assess the effects of NMMII on vesicle dynamics, synaptic vesicle mobility was measured using FRAP under conditions of altered Myosin activity. To visualize synaptic vesicles *in vivo*, the recombinant *synaptotagmin-GFP *construct, *elav*^*C155*^*Gal4; UAS-sytGFP*, was used to drive expression of *synaptotagmin-GFP *in the nervous system. *Synaptotagmin-GFP *has previously been shown to mark synaptic vesicles [12,24]. To begin our examination of myosin activity at the synapse we used ML-9, an inhibitor of myosin light chain kinase (MLCK), and tested synaptic vesicle mobility under different doses of this Myosin inhibitor (Figure 3A). Application of ML-9 to *elav*^*C155*^*Gal4; UAS-sytGFP/Y *larvae resulted in a dose-dependent reduction in the mobility of synaptic vesicles compared to vesicle dynamics in a saline + DMSO control (Figure 3B). Vesicle mobility in this saline + DMSO treated control (n = 20 boutons, 4 larvae) did not significantly differ from the saline only control (n = 22 boutons from 4 larvae, P > 0.05) used here in the subsequent FRAP experiments. A significant decrease in vesicle mobility was observed with 100 μM ML-9, which resulted in the most dramatic reduction of vesicle mobility (n = 20 boutons from 4 larvae, P < 0.05) while 50 μM ML-9 resulted in slightly, but significantly less vesicle mobility (n = 20 boutons from 4 larvae, P < 0.05) compared to the control. A dose of 10 μM ML-9 (n = 20 boutons from 4 larvae, P > 0.05) had no effect on vesicle dynamics (Figure 3B, Table 1 and Additional files 1 and 2 for example movies of vesicle dynamics with control and 100 μM ML-9). Together this indicates a dose-dependent response of vesicle mobility to Myosin inhibition. ML-9 is a general Myosin inhibitor while (-)-Blebbistatin is known to be selective towards inhibiting NMMII [25]. However (-)-Blebbistatin is autofluorescent under blue light and is quickly deactivated upon exposure to blue light [26,27] thus limiting our ability to use this drug with our FRAP conditions. We did however obtain NMMII alleles to determine their affect on vesicle mobility.

**Table 1:** Rate parameters from FRAP curves fit with the double exponential curves

Treatment	Rate Parameters^1^			
	**A**	**K1**	**B**	**K2**

*Vesicle Mobility*				

				

0 μM ML-9	0.38 ± 0.031	0.0040 ± 0.0010	0.72 ± 0.031	0.11 ± 0.40

10 μM ML-9	0.37 ± 0.0035	0.0035 ± 0.0010	0.40 ± 0.083	0.080 ± 0.023

50 μM ML-9	0.39 ± 0.030	0.0025 ± 0.0010	0.42 ± 0.087	0.080 ± 0.022

100 μM ML-9	0.51 ± 0.020	0.0025 ± 0.0005	0.29 ± 0.16	0.11 ± 0.050

				

*Control (ct)*	0.40 ± 0.033	0.0047 ± 0.0012	0.58 ± 0.22	0.10 ± 0.039

*Heterozygous Loss-of-Function (Het)*	0.37 ± 0.043	0.0069 ± 0.0015	0.66 ± 0.33	0.12 ± 0.049

*RNAi Knockdown (K/D)*	0.46 ± 0.035	0.0031 ± 0.0008	0.28 ± 0.12	0.082 ± 0.045

*Overexpression (O/E)*	0.49 ± 0.040	0.0023 ± 0.0010	0.35 ± 0.093	0.071 ± 0.030

^1 ^Equation: Frap(t) = 1-A(e^-K1 off t^)-B(e^-K2 off t^) [38] where A, B = constants that represent the apparent bleached fraction, K1, K2 = rates, t = time

### Nonmuscle Myosin II contributes to synaptic vesicle dynamics

**Figure 4:**
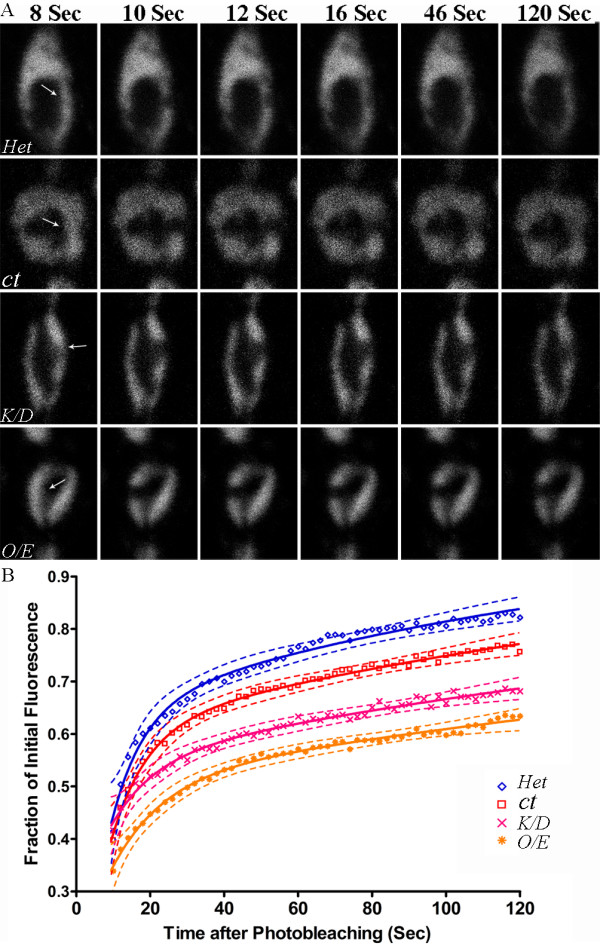
**FRAP recovery curves for vesicle mobility when NMMII expression is altered**. *a*, Acquired images for the recovery of the heterozygous loss-of-function, *elav*^*C155*^*Gal4;UAS-sytGFP/+; zip*^*1*^*/+ *(*Het*), the control, *elav*^*C155*^*Gal4;UAS-sytGFP/Y *(*ct*), the RNAi knockdown, *elav*^*C155*^*Gal4;UAS-sytGFP/+;UASzipRNAi/+ *(*K/D*) and the overexpression, *elav*^*C155*^*Gal4;UAS-sytGFP/+; zip*^*GS50077*^*/+ *(*O/E*) of NMMII immediately before bleaching (8 sec), immediately after photobleaching (10 sec) and at 12, 16, 48 and 120 sec post-bleaching. Bleached areas are indicated with white arrows. At 16 sec the bleached region for *Het *is no longer visible; however, it is still clearly visible in control (*ct*). At 120 sec, the bleached region is no longer visible in the control (*ct*), but is still visible for both *K/D *and *O/E*. *b*, Vesicle mobility is affected by the expression level of NMMII. FRAP curves reveal that the heterozygous NMMII loss-of-function (*Het*) significantly enhanced vesicle mobility as compared to the control (*ct*) while both knockdown (*K/D*) and overexpression (*O/E*) of NMMII significantly reduced vesicle mobility. FRAP recoveries were fit with double exponential curves. Nonlinear regression was used to test for statistical differences; *elav*^*C155*^*Gal4;UAS-sytGFP/*Y (n = 26), *elav*^*C155*^*Gal4;UAS-sytGFP/+; zip*^*1*^*/+ *(n = 24, p < 0.05), *elav*^*C155*^*Gal4;UAS-sytGFP/+;UASzipRNAi/+ *(n = 22, p < 0.05), *elav*^*C155*^*Gal4;UAS-sytGFP/+; zip*^*GS50077*^*/+ *(n = 20, p < 0.05).

In order to clarify whether NMMII was contributing to the inhibition of vesicle dynamics by ML-9, we genetically combined *elav*^*C155*^*Gal4;UAS-sytGFP *with the NMMII alleles; *zip*^*1*^, *UASzipRNAi *and *zip*^*GS50077*^. This allowed us to visualize the affect on vesicle dynamics when NMMII expression was altered (Figure 4A). When NMMII levels are knocked down using *elav*^*C155*^*Gal4;UAS-sytGFP/+; UASzipRNAi/+ *(*K/D*, n = 26 boutons from 5 larvae) the rate of vesicle movement is significantly reduced compared to the *elav*^*C155*^*Gal4;UAS-sytGFP/Y *(*ct*, n = 19 boutons from 4 larvae, P < 0.05) control. Surprisingly, vesicle dynamics were also reduced (*O/E*, n = 23 boutons from 5 larvae, P < 0.0001) when NMMII is overexpressed using *elav*^*C155*^*Gal4;UAS-sytGFP/+; zip*^*GS50077*^/+. Interestingly, the heterozygous loss-of-function NMMII allele, *zip*^*1*^*/+ *resulted in enhanced mobility of vesicles (*Het*, n = 21 boutons from 4 larvae, P < 0.05) (Figure. 4B, Table 1 and Additional files 1, 3, 4 and 5 for example movies of vesicle dynamics with NMMII manipulation).

Although the bleach depth was not significantly different between the heterozygous loss-of-function allele, the RNAi knockdown sample and control samples, the O/E samples did show significantly lower bleach depth (See Additional file 6, Figure S1A showing the average bleach depth from FRAP experiments). We therefore further analysed the FRAP recoveries to ensure differences in bleach depth did not account for our results. To refine our analysis, we calculated the recovery index of the FRAP curve [12] which accounts for differences in bleach depth between genotypes. These calculations led to the same conclusions: the heterozygous loss-of-function allele of NMMII increased the recovery index, while both the RNAi knockdown and overexpression of NMMII reduced the recovery index (See Additional file 6, Figure S1B showing the recovery index from the FRAP curves).

Overall these results indicate that NMMII contributes to vesicle mobility in a manner which is highly dependent on the expression level of NMMII (Table 2).

**Table 2:** Summary of results from manipulating NMMII expression

NMMII Allele	Expression of NMMII	Vesicle Mobility
		

*O/E*	↑	↓

		

*Het*	↓	↑

		

*K/D*	↓↓	↓

↑ = increased relative to control, ↓ = decreased relative to control, ↓↓ = further decreased relative to control

## Discussion

This report has identified the presence of NMMII in the presynaptic terminal and indicates a function for NMMII in synaptic vesicle mobility at the NMJ of *Drosophila melanogaster*. NMMII has been implicated in synaptic transmission in rats [19], but has not previously been shown at the NMJ of *Drosophila *and this is the first evidence of NMMII having a function in synaptic vesicle mobility. Using *Drosophila*, as a genetically malleable tool, and the confocal imaging technique, FRAP, we were able to quantify the effect of NMMII on vesicle mobility. FRAP revealed that NMMII plays a complex role in vesicle dynamics and begins to clarify our knowledge of how synaptic vesicles may be available for release upon stimulation.

### Vesicle mobility is affected by a complex interaction with Nonmuscle Myosin II

Immunocytological staining first identified that NMMII is found both pre- and postsynaptically in the *Drosophila *NMJ. Abolishment of postsynaptic NMMII expression through RNAi confirmed expression of NMMII presynaptically. With NMMII present in the presynaptic terminal, this suggests a possible function for NMMII in trafficking vesicles within the bouton. To determine whether NMMII impacts vesicle dynamics, we carried out *in vivo *imaging techniques to visualize synaptic vesicle mobility. We found an intriguing complex interaction between the expression level of NMMII and the dynamics of vesicle mobility. Inhibiting MLCK reduced vesicle mobility, consistent with Jordan et al. [10]. More specifically, increasing either NMMII expression by 95% or reducing NMMII expression to 28% reduced vesicle mobility, while moderately reducing NMMII to 57% enhanced vesicle mobility. A limitation of the present study is an estimate of NMMII activity or levels specifically at nerve terminal boutons in the various mutant strains used. The postsynaptic presence of NMMII makes immunohistochemical techniques for measuring presynaptic NMMII difficult. We estimated neuronal NMMII levels using western blots of larval brains. This demonstrated that NMMII levels were up- and down-regulated significantly by the genotypes used. The 50% reduction in protein level from the heterozygous loss-of-function to the RNAi knockdown strains did not exhibit a statistical difference. However, we did find substantially different effects on vesicle mobility suggesting that the apparent difference in expression levels is functionally significant. Thus it appears that tight regulation of NMMII expression is essential in maintaining appropriate vesicle dynamics: a small reduction in NMMII levels enhances mobility whereas too much or too little impairs mobility. Together this supports a role for NMMII in normal synaptic vesicle mobility at the *Drosophila *NMJ.

While these findings suggest a role for NMMII in synaptic vesicle mobility, it does not exclude the possibility that other myosin motors are also involved. In support of this, the general myosin inhibitor, ML-9, reduced synaptic vesicle mobility in a dose dependent manner with no stimulation of vesicle mobility at low concentrations. In addition, other myosin motors have been shown to associate with synaptic vesicles and be involved in synaptic transmission. For example, Myosin V can bind to a myosin receptor found on a subpopulation of high density vesicles [28] and is found to be associated with vesicles isolated from chick brain [29], but was not found to alter hippocampal synaptic transmission in mice [30]. However, Myosin II has been associated with normal synaptic transmission. In cultured rat superior cervical ganglion neurones, myosin IIb was found to inhibit synaptic transmission [19]. A reduction in synaptic transmission upon inhibition of Myosin II was also observed in rat cholinergic synapses [31], while MLCK was found to be involved in maintaining repetitive synaptic transmission [32]. Myosin II has also been shown to be involved in vesicle mobility in other systems. Nonmuscle Myosin II has been shown to transport vesicles on actin filaments in clam oocytes [22] and Nonmuscle Myosin II has been shown to contribute to vesicle transport from the Golgi to the Endoplasmic Reticulum [23]. Thus, while the present work identifies a function for NMMII in synaptic vesicle mobility, the precise mechanisms of myosin motors in synaptic transmission and the precise role of NMMII as a vesicle motor remains to be clarified. Since NMMII interacts with actin, it will also be important to investigate the affects of NMMII on actin stability and dynamics in the NMJ and to determine whether the affects of NMMII on vesicle mobility translate into affects on synaptic transmission.

## Conclusions

Our results show that NMMII is found presynaptically at the *Drosophila *NMJ and plays a functional role at the NMJ. We report, for the first time, a function for NMMII in normal synaptic vesicle mobility in the unstimulated neuron, which is dependent on the expression level of NMMII. Further experimentation to address the function of NMMII at the NMJ, through electrophysiological assays, manipulating NMMII activity by its' kinases and phosphatases and measuring actin activity, is required to more clearly define the precise role of NMMII at the *Drosophila *NMJ.

## Methods

### Drosophila Stocks

*Drosophila melanogaster *were maintained at 22°C on Bloomington fly media. The heterozygous loss-of-function NMMII allele, *zipper*^1^*/CyO *(*zip*^1^*/CyO*) (FBal0018862) was acquired from the Bloomington stock center and rebalanced over *Cyo-GFP*. *UASzipperRNAi *(*UASzipRNAi*) was obtained from the Vienna *Drosophila *RNAi center (FBst0470845) and is a NMMII RNAi construct. The gain-of-function NMMII construct, *zipper*^*GS50077*^(*zip*^*GS50077*^), was obtained from the *Drosophila *gene search project [33] and is a unidirectional UAS construct inserted upstream of NMMII. *elav*^*C155*^*Gal4; UASsynaptotagminGFP *(*elav*^*C155*^*Gal4;UAS-sytGFP*) was obtained from the Bloomington stock center (FBst0006923) and *UASactinGFP *(*UAS-actGFP*) was obtained from the Kyoto stock center for FRAP imaging of vesicle and actin dynamics respectively. A stock of *UAS-actGFP/UAS-actGFP; elav*^*3A*^*Gal4/TM3, Sb, Tb *was generated to express actinGFP in the nervous system. The Gal4 drivers, *elav*^*3A*^*Gal4 *(FBti0072910, Bloomington stock center) and *elav*^*C155*^*Gal4 *drive expression of UAS constructs in the nervous system [34]. *24BGal4 *(FBti0002090, Bloomington stock center) was used to drive expression of NMMII alleles in muscle. Flies were crossed at 25°C and kept at identical growing conditions.

### Immunocytochemistry

Third instar larva were dissected in HL3 buffer with no Ca^2+ ^added [35], and fixed in 4% paraformaldehyde in phosphate-buffered saline (PBS). After fixation, the samples were transferred to 1% BSA diluted in PBT (PBS plus 0.1% Triton X-100). The following primary antibodies were used: rabbit anti-NMMII (1:1000, Roger Karess, Centre de National de Récherche Scientifique, Centre de Génétique Moléculaire), and mouse anti-Dlg (1:200, Developmental Studies Hybridoma Bank). Anti-HRP FITC (1:1000, MP Biomedical; Solon, OH) was used as a neural marker. The secondary antibodies used were; goat anti-mouse Alexa488 and goat anti-rabbit Alexa633 (1:1000, Invitrogen). Following antibody labelling, the preparations were washed in PBT and mounted in Vectashield (Vector Laboratories; Burlington, ON).

Images were acquired on a Carl Zeiss LSM510 confocal. Z-sections were obtained on a F-Fluar 40×/1.3 Oil for NMMII localization. The pinhole aperture acquired images of 1 μm thickness and image sections were projected onto a single plane. 1-3 images were collected per larva.

### Western blot

For each treatment, five brains from third instar larvae were lysed in protein lysis buffer (50 mM Tris-Cl, 1% NP-40, 150 mM NaCl) with complete protease inhibitor. Samples were separated on a 10% SDS-polyacrylamide gel, and transferred to PVDF membrane. NMMII was detected with rabbit anti-NMMII antibody (1:10000, Roger Karess, Centre de National de Récherche Scientifique, Centre de Génétique Moléculaire), and a goat anti-rabbit HRP-coupled secondary (1:1500 BioRad). α-β-Tubulin was used as a loading control (1:100, Developmental Studies Hybridoma Bank). Antibodies were visualized using chemiluminescent detection (ECL Plus, Amersham). Control and experimental bands were imaged either simultaneous or individually on the same blot. Blots were scanned and digitized with a Molecular Dynamics Phosphoimager. Band intensities were quantified using ImageJ.

### Fluorescence Recovery After Photobleaching

FRAP was conducted on a Carl Zeiss LSM 510 confocal microscope equipped with an Argon2 laser and a LP505 filter. To immobilize the preparation, wandering third instar larvae were dissected in HL3 with no Ca^2+ ^added [35] and glued to slygard-coated slides using Nexabrand tissue glue (WPI, Sarasota, FL). The glued preparations were placed under an Achroplan 100×/1.0 W Ph objective with an 8× digital zoom. FRAP recordings were made, from segments 3 or 4, of type I boutons at muscle 7/6 for vesicle dynamics. Images were collected at 1.12 *μ*s/pixel with a pinhole of 1 airy unit and a resolution of 512 × 512. Sixty images were collected over two minutes with a 1 sec delay between image acquisitions. To select the area for bleaching, a region of interest (ROI) 24 × 30 pixels was selected on the digital image. Four baseline scans were acquired using 5% or 10% of full laser power. Before the fifth scan, the laser increased to 97% of maximal and rapidly iterated the ROI 9 times, after which, returning to 5% or 10% of maximal power to complete the remaining 56 scans. A maximum of three type I boutons were recorded per hemi-segment and a maximum of 6 boutons per larvae. All FRAP experiments were completed within 2 hours of the larval dissection.

The acquired image sequences were imported into ImageJ. Images that drifted were digitally stabilized before analysis [36]. The time series analyzer plug-in for ImageJ was used to measure fluorescent intensity. Intensity measurements were taken from the background, the total fluorescence in the bouton, and the fluorescence in the bleached area. A double normalization was completed, as described in [37] using the following equation:

Where T = total fluorescence in the bouton, I = fluorescence in the bleached fraction and BG = background fluorescence outside the bouton. This accounts for photobleaching throughout the FRAP experiment and for movement of bleached molecules out of the FRAP ROI.

The recovery curves were fit two a double exponential curve [38] as follows:

Where A, B = constants that represent the apparent bleached fraction, K1, K2 = rates, t = time.

### Drug administration

To access vesicle dynamics under myosin inhibition, the myosin light chain kinase inhibitor, ML-9, was applied to the preparation before beginning FRAP experiments. A stock concentration of 50 mM ML-9 in DMSO was diluted into HL3 [35] with no Ca^2+ ^added, to make 100 μM, 50 μM and 10 μM ML-9 solutions. The preparations were incubated in the dark with ML-9 for 30 minutes prior to FRAP.

### Statistical analysis

All statistical analyses were performed in GraphPad Prism 4.0. For non-linear regression, FRAP was double normalized [37] and compiled for curve fitting [38]. In all cases, the double exponential curve outlined in McNally (2008) [38] was accepted over the single exponential curve. Error is represented as the 95% confidence interval for the curve. Rates are expressed as inverse seconds. In double exponential curves, the initial phase has been indicated here with A and K1 and the second phase of the curve have been indicated by B and K2. For analysis of variance, one-way ANOVA was completed. P < 0.05 was accepted as statistically significant.

## Authors' contributions

SS performed and analysed all FRAP experiments, contributed to conceiving and designing the studies and prepared the manuscript. XQ carried out and analysed western blot data, completed the immunocytochemistry and contributed to the design of the studies. BAS conceived and assisted in designing and conducting the studies, provided feedback and editing of the manuscript and supplied materials and reagents which made these studies possible. All authors read and approved the final manuscript.

## Supplementary Material

Additional file 1 **Movie 1.** FRAP of synaptic vesicles labelled with *synaptotagmin-GFP*. Time-lapse video recorded over two minutes and compressed into 9 seconds. Click here for file

Additional file 2 **Movie 2.** FRAP of labelled synaptic vesicles whose mobility has been inhibited with application of 100 μM ML-9. Time-lapse video recorded over two minutes and compressed into 9 seconds. Click here for file

Additional file 3 **Movie 3.** FRAP of labelled synaptic vesicles showing that mobility is reduced with RNAi knockdown of NMMII. Time-lapse video recorded over two minutes and compressed into 9 seconds. Click here for file

Additional file 4 **Movie 4.** FRAP of labelled synaptic vesicles whose mobility is enhanced with a moderate reduction of NMMII. Time-lapse video recorded over two minutes and compressed into 9 seconds. Click here for file

Additional file 5 **Movie 5.** FRAP of *synaptotagmin-GFP *labelled vesicles showing reduced vesicle mobility with overexpression of NMMII. Time-lapse video recorded over two minutes and compressed into 9 seconds. Click here for file

Additional file 6 **Figure S1.** Graphs indicating the average bleach depth and the recovery index for the NMMII FRAP experiments. Click here for file
