# Supplementary material for: Nonmuscle Myosin II helps regulate synaptic vesicle mobility at the Drosophila neuromuscular junction
Source: BMC Neurosci. 2010 Mar 16;11:37. doi: 10.1186/1471-2202-11-37 (PMC2853426; doi:10.1186/1471-2202-11-37)
Supplement: Additional file 6 — Figure S1. Graphs indicating the average bleach depth and the recovery index for the NMMII FRAP experiments. [file 1471-2202-11-37-S6.PDF]

Supplementary Figure 1

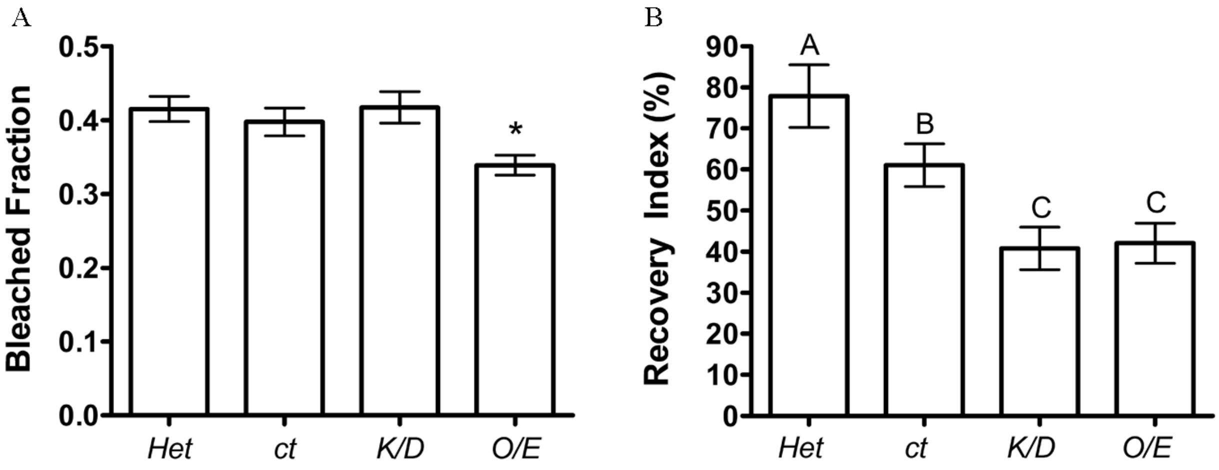

Further analysis of NMMII FRAP recoveries. A, Bleach depth average of each genotype. The heterozygous loss-of-function (*Het,* 0.42 ± 0.08, n=21 boutons from 4 larvae), the control (*ct,* 0.40 ± 0.08, n=19 boutons from 4 larvae) and the NMMII RNAi knockdown (*K/D*, 0.42 ± 0.11, n=26 boutons from 5 larvae) did not differ significantly in their bleach depth (P>0.05). Overexpression (*O/E,* 0.34 ± 0.06, n=23 boutons from 5 larvae) exhibited a significantly lower bleach depth. (P<0.05). B, The recovery index (RI) was calculated for each genotype using the following equation:

$$RI = \frac{Fp - Fb}{Fi - Fb}$$

Where Fp = the peak fluorescence for each recovery curve, Fb = the post-bleaching intensity in the bleached ROI and Fi = the initial fluorescence in the bleached ROI. This accounts for differences in photobleaching depth. The heterozygous loss-of-function NMMII allele (*Het*, 77.89 ± 37.37%, n=24 boutons from 4 larvae, P<0.05) exhibited a significantly greater RI than the control (*ct,* 61.06 ± 24.55%, n=22 boutons from 4 larvae) while both the overexpression (*O/E*, 42.06 ± 23.34%, n=23 boutons from 5 larvae, P<0.05) and RNAi knockdown (*K/D*, 40.79 ± 26.34%, n=26 boutons from 5 larvae, P<0.05) of NMMII exhibited significantly lower RIs which is consistent with the results obtained using a double exponential curve fit described in McNally (2008). Similar letters indicate peaks which are statistically the same. * indicates P<0.05. P<0.05 was taken as significantly different.
